# Supplementary material for: Splice-Junction-Based Mapping of Alternative Isoforms in the Human Proteome
Source: Cell Rep. Author manuscript; Available in PMC 2020 Jan 15. (PMC6961840; doi:10.1016/j.celrep.2019.11.026)
Supplement: 3 [file NIHMS1546469-supplement-3.zip › DF2/PXD0010154/Thyroid-175-Q12805-RGEQCVDIDECR.pdf]

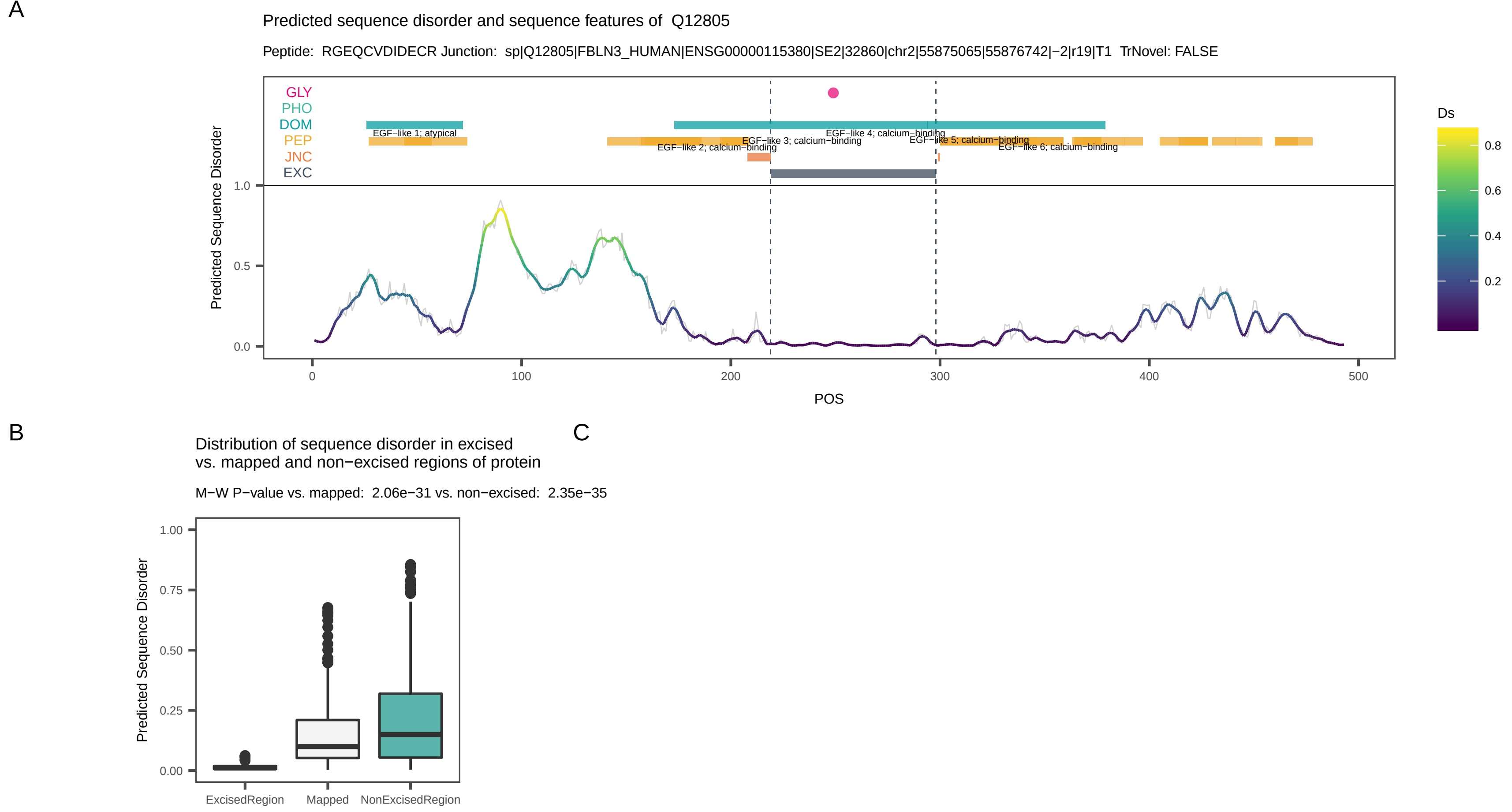
A
Predicted sequence disorder and sequence features of Q12805
Peptide: RGEQCVDIDECR Junction: sp|Q12805|FBLN3_HUMAN|ENSG00000115380|SE2|32860|chr2|55875065|55876742|−2|r19|T1 TrNovel: FALSE
GLY
PHO
DOM
PEP
JNC
EXC
EGF−like 1; atypical
EGF−like 2; calcium−binding
EGF−like 3; calcium−binding
EGF−like 4; calcium−binding
EGF−like 5; calcium−binding
EGF−like 6; calcium−binding
Predicted Sequence Disorder
1.0
0.5
0.0
0
100
200
300
400
500
POS
Ds
0.8
0.6
0.4
0.2
B
Distribution of sequence disorder in excised
vs. mapped and non−excised regions of protein
M−W P−value vs. mapped: 2.06e−31 vs. non−excised: 2.35e−35
Predicted Sequence Disorder
1.00
0.75
0.50
0.25
0.00
ExcisedRegion
Mapped
NonExcisedRegion
C
